# An Interesting Case of Cellulitis Caused by Shewanella

**DOI:** 10.7759/cureus.9719

**Published:** 2020-08-13

**Authors:** Ali Hussain, Mohsin Gondal, Hira Yousuf, Jan Ganai, Muhammad Junaid Mahboob

**Affiliations:** 1 Acute Medicine, Pinderfields General Hospital, Wakefield, GBR; 2 Cardiology, Sheffield Teaching Hospitals NHS Foundation Trust, Sheffield, GBR; 3 Medical Oncology, Pinderfields General Hospital, Wakefield, GBR; 4 Internal Medicine, Pinderfields General Hospital, Wakefield, GBR; 5 Internal Medicine, Jinnah Postgraduate Medical Centre, Karachi, PAK

**Keywords:** case report, shewanella, marine water, cellulitis

## Abstract

*Shewanella* species are opportunistically pathogenic, gram-negative bacilli that are part of marine microflora. Infection caused by *Shewanella* species in humans is rare and mostly acquired after direct contact with seawater or ingestion of raw seafood. The exact pathogenesis remains unclear. Cutaneous infections are among the most common manifestation with underlying skin diseases and immune-compromised states; however, bacteremia from lungs, abdominal, and biliary sepsis has also been reported. These infections are difficult to diagnose due to limited physicians' experience and scarce microbiological data available. Hence, delayed diagnosis and treatment could be fatal and may result in sepsis with multi-organ failure. Our case report reiterates the fact that careful attention should be devoted to unusual circumstances in history and atypical pathogens on cultures if there is no or minimal clinical improvement after antibiotics.

## Introduction

*Shewanella* is a group of opportunistic organisms that mainly causes infection in immunocompromised individuals. Initially, these species were named *Achromobacter putrefaciens*, later changed to *Pseudomonas putrefaciens*, and was finally, in 1985, classified as *Shewanella* genus. There are approximately 30 *Shewanella* strains; however, infections in humans are mainly caused by* S. algae* and* S. putrefaciens* [1]. Most widely reported presentations include skin and soft tissue infections. A careful history of marine water exposure is vital in deciphering *Shewanella* infection in vulnerable patients as delay in diagnosis could be catastrophic. We, herein, report a case of fulminant cellulitis caused by *Shewanella* in a multi-morbid patient after swimming in ocean water following a ritual belief that it improves the skin conditions. The patient had a timely diagnosis with excellent recovery following a culture-directed antibiotic therapy and was advised strictly against any future exposure to marine waters.

## Case presentation

The patient is a 75-year-old male, known case of long-standing type II diabetes mellitus and extensive psoriasis. He had a history of chronic kidney disease (stage 3) secondary to diabetes and was under regular follow-up with the nephrology team. He had diabetic foot ulcers, which were healing slowly due to peripheral vascular disease and diabetic neuropathy. In addition, he also had chronic dependent leg edema due to limited mobility.

He presented to the emergency department (ED) with a history of feeling generally unwell, tired, and acute confusion. About 48 hours prior to attending the ED, he developed a generalized intractable itching, for which he was treated as exacerbation of psoriasis, and steroid treatment was initiated by his dermatologist. Within 48 hours of starting steroids, he deteriorated and was brought to the ED.

**Figure 1:**
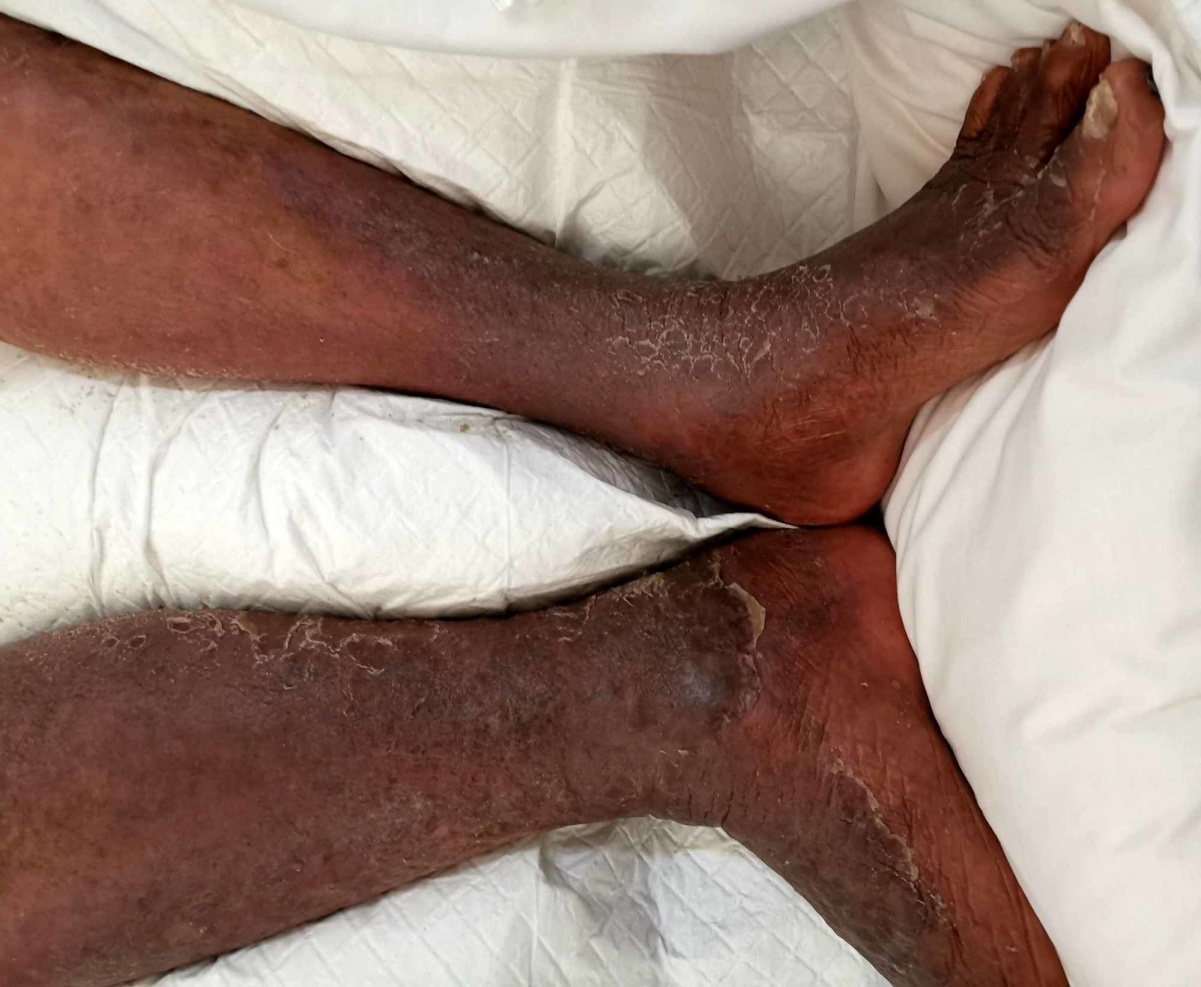
Cellulitis of both legs with skin thickening, ruptured bullas, and erythema.

On arrival to the ED, his vitals were as follows: blood pressure of 145/80 mmHg, pulse rate of 140 beats/minute, temperature of 39, respiratory rate of 22 breaths/minute, and his blood sugar by finger stick was undetectable (due to high values). On subsequent serum sample, it turned out to be 40 mmol/L. On examination, he was drowsy but responsive to voice and had flapping tremors with myoclonic jerks. There was no focal neurological deficit. He had generalized redness of the skin, suggestive of erythrodermic psoriasis. His leg examination showed (Figure 1) bullous weeping lesions, skin thickening, and erythema. There was tenderness of lower limbs on palpation; however, no crepitus was noted. Distal pulses were not palpable on clinical examination.

On routine biochemistry (Table 1), he was found to have hyperglycemic hyperosmolar syndrome (HHS) and was started on treatment according to the local HHS protocol. His renal functions also showed marked worsening (stage 3 acute renal failure) along with hyperkalemia (K 6.4 mmol/L), but no obvious tall tented T-waves were present.

**Table 1:** Laboratory parameters for a patient on admission. AKI, acute kidney injury; ALT, Alanine aminotransferase; AST, aspartate aminotransferase

Parameters	Normal range (units)	Patient results (on admission)
Hemoglobin	11.0-14.5 g/dL	10.5
Mean corpuscular volume	78.0-95.0 fL	85.2
White blood count	2.4-9.5 x 10^9^/L	17.6
Neutrophils	1.0-5.0 x 10^9^/L	15.0
Lymphocytes	1.2- 4 x 10^9^/L	2.4
Eosinophils	0.15- 0.5 x 10^9^/L	0.1
Monocytes	0.0-0.2 x 10^9^/L	0.1
Basophils	5 x 10^9^/L	0.0
Platelets	150-450 x 10^9^/L	239
Urea	2.8-8.1 mmol/L	42.7
Creatinine	45-84 µmol/L	307
AKI	0	3
Sodium	135-145 mmol/L	138
Potassium	3.5-5.1 mmol/L	6.4
Chloride	98-107 mmol/L	104
Bicarbonate	22-29 mmol/L	15
­pH (venous gas)	7.33-7.44	7.32
Anion gap (venous gas)	5-13 mmol/L	15
Random blood glucose (serum)	4.1-5.9 mmol/L	40
Ketones (on strip)	0 (absent)	1
Osmolality (serum)	275-295 mOsmol/kg	337
ALT	0-33 U/L	25
AST	0-33 U/L	20
Albumin	35-52 g/L	30
Bilirubin	0-17 µmol/L	10

Deep vein thrombosis and limb ischemia were ruled out by Duplex ultrasonography of the legs. Plain X-rays of lower limbs excluded gas gangrene. He was seen by the plastic surgery team, and necrotizing fasciitis was excluded from the above corroborative detailed investigations.

The patient had an initial presumptive diagnosis of “Sepsis secondary to cellulitis along with Acute-on-Chronic kidney injury and HHS”. He was started on sepsis protocol, and flucloxacillin 1 gm (every six hours) was commenced after blood cultures drawn. Plain CT of the head (non-contrast) was performed, which did not show any intracranial cause of acute delirium.

In the next 48 hours, there was a slight improvement in his clinical condition and infective markers but continued to spike temperatures. After 72 hours, we received the results of blood cultures from the microbiologist, which, to our surprise, were positive for *Shewanella *algae. Retrospectively, patient history was revisited in which he had disclosed taking a bath in marine water, as there was a local belief that seawater cures psoriasis.

His antibiotics were changed from flucloxacillin to tazobactam-piperacillin 4.5 gm (in renal dose) according to blood culture sensitivity testing and had a remarkable improvement in clinical status and infection markers. Furthermore, his renal functions also recovered back to his baseline levels along with the resolution of HHS. The patient was discharged home after seven days of intravenous antibiotics, and repeat blood cultures were negative. Moreover, the patient was given strict advice regarding any further exposure to marine water.

## Discussion

*Shewanella* is a facultative, non-fermentative gram-negative anaerobe. Ecologically, it not only inhabits all forms of water and soil but is also isolated from fish, dairy products, oils, and animal carcasses. However, it rarely causes infections in humans [2]. The modes of infection reported so far in different case reports are contact with the marine water or consumption of seas foods [3]. The common infections caused by *Shewanella* are shown in Table 2.

**Table 2:** Human infections caused by Shewanella [3].

Skin and soft tissue infections
Ear infection (otitis media)
Eye infection
Infective arthritis
Osteomyelitis
Infective endocarditis
Biliary tract infections and peritonitis

The exact pathogenesis of this infection remains unknown. Both host factors (Table 3) and the ability of *Shewanella* to produce hemolysin are responsible. However, in organ-specific infections, for example, lung infections are linked to exposure to marine water in near-drowning or head submerging during recreational seawater activities [4]. Likewise, Vignier et al. had reported *Shewanella* in abdominal and biliary tract infections [5]. Skin and soft tissue infections are commonly associated with breaches in the skin such as ulcers or following trauma, as in our case, in which the patient had a fungal infection that provided a portal of entry for bacteria.

**Table 3:** Risk factors for Shewanella infection.

Factors
Host factor
Chronic leg ulcer
Peripheral vascular occlusive disease
Diabetes mellitus
Chronic liver and kidney diseases
Skin disease
Pathogen factor
Exposure to seawater
Hemolysin

Treatment

Management of most *Shewanella* infections includes combinations of surgical therapy (debridement or drainage) and antibiotics [6-7]. It is noteworthy that poor outcome is usually associated with underlying disease [8]. In particular, there are no specific antibiotics guidelines or protocols for *Shewanella*-associated infections. Therefore, local microbiologist advice should be sought and followed. Since *Shewanella* is oxidase-positive, most laboratories tend to perform sensitivity for broad-spectrum antibiotics. Vignier et al. [5] reviewed the in vitro antimicrobial susceptibility of *Shewanella* and found that most isolates were sensitive to third-generation cephalosporin, tazobactam/piperacillin, ciprofloxacin, and gentamicin. In terms of resistance, it showed a propensity toward carbapenems [9]. Mechanism of resistance may be related to carbapenem hydrolyzing amber class D β-lactamase [10].

## Conclusions

Our case report further consolidates the correlation of *Shewanella* infection with exposure to marine water. It also emphasizes that people with underlying skin diseases are more susceptible to infections and hence all possible efforts should be made to avoid having any skin breach to prevent portal of entry to opportunistic organisms. As in our reported case, the patient’s underlying psoriasis paved the path for *Shewanella* infection, and hence patient education is vital to prevent such future events.
